# Deep convolutional neural network-based skeletal classification of cephalometric image compared with automated-tracing software

**DOI:** 10.1038/s41598-022-15856-6

**Published:** 2022-07-08

**Authors:** Ho-Jin Kim, Kyoung Dong Kim, Do-Hoon Kim

**Affiliations:** 1 Department of Orthodontics, School of Dentistry, Kyungpook National University, 2175, Dalgubul-Daero, Jung-Gu, Daegu, 41940 Korea; 2 School of Electronic and Electrical Engineering College of IT Engineering, Kyungpook National University, Daegu, Korea; 3 Medical Big Data Research Center, Kyungpook National University, Daegu, Korea

**Keywords:** Orthodontics, Electrical and electronic engineering

## Abstract

This study aimed to investigate deep convolutional neural network- (DCNN-) based artificial intelligence (AI) model using cephalometric images for the classification of sagittal skeletal relationships and compare the performance of the newly developed DCNN-based AI model with that of the automated-tracing AI software. A total of 1574 cephalometric images were included and classified based on the A-point-Nasion- (N-) point-B-point (ANB) angle (Class I being 0–4°, Class II > 4°, and Class III < 0°). The DCNN-based AI model was developed using training (1334 images) and validation (120 images) sets with a standard classification label for the individual images. A test set of 120 images was used to compare the AI models. The agreement of the DCNN-based AI model or the automated-tracing AI software with a standard classification label was measured using Cohen’s kappa coefficient (0.913 for the DCNN-based AI model; 0.775 for the automated-tracing AI software). In terms of their performances, the micro-average values of the DCNN-based AI model (sensitivity, 0.94; specificity, 0.97; precision, 0.94; accuracy, 0.96) were higher than those of the automated-tracing AI software (sensitivity, 0.85; specificity, 0.93; precision, 0.85; accuracy, 0.90). With regard to the sagittal skeletal classification using cephalometric images, the DCNN-based AI model outperformed the automated-tracing AI software.

## Introduction

In the field of orthodontics, accurate diagnosis is of clinical importance because it is closely associated with treatment planning and subsequent outcomes. Among clinical parameters for diagnosis, the A-, Nasion- (N-), and B-points (ANB) angle is generally measured on lateral cephalometric images to evaluate the sagittal skeletal relationship that is closely related to occlusal relationship and facial appearance. Based on the ANB angle, the patients can be categorized as having skeletal Class I, II, and III relationships, which may affect the decision-making in treatment planning.

Recently, artificial intelligence- (AI-) based diagnosis has been performed in the treatment planning, increasingly drawing the attention of orthodontists. In 1956, computer scientist John McCarthy defined AI as the science and engineering of making highly intelligent computing machines or computer programs. Recently, as a part of AI and machine learning, deep learning algorithms, including deep convolutional neural network (DCNN), recurrent neural network (RNN), generative adversarial network (GAN), and deep belief network (DBN), have been popularly used in numerous fields. Particularly, the DCNN systems have demonstrated high performance in image analysis and recognition and in the process of extracting image characteristics and learning their patterns. Regarding deep-learning-based diagnosis in medicine, several studies have reported that the DCNN also displays superior abilities when applied to medical images^1,2^.

In terms of orthodontic analysis and diagnosis, research is being increasingly conducted on DCNN systems based on dental x-ray images. Moreover, several software methodologies based on their own specific AI algorithms are already being effectively used^3,4^. There are two issues in deep learning studies using the cephalogram. First, automated detection of landmarks is a popular diagnosis issue. Hwang et al.^5^ reported that AI detected 19 cephalometric landmarks accurately with a mean detection error of < 2 mm. Regarding the differences in cephalometric measurements between an orthodontist and AI, a previous study mentioned that the measurement error of AI is clinically acceptable^3^. Second, direct classification or analysis using cephalometric image-based DCNN algorithms is another popular issue. Contrary to the automated-tracing AI model, this method can eliminate the steps in detecting landmarks and in the interpretation of the cephalometric measurements. Thus, immediate image-oriented diagnosis is achieved in the decision-making process by minimizing the errors in diagnosis and treatment planning by decreasing the number of steps. Previous studies have reported skeletal classification and differential diagnosis in the extraction of teeth or surgery with an accuracy > 90% based on DCNN-based deep learning^6–8^.

Therefore, this study aims to investigate the DCNN-based AI model using cephalometric images for the classification of sagittal skeletal relationships and compare the performance of the newly developed DCNN-based AI model with that of the automated-tracing AI software.

## Methods

This research was approved by the Institutional Review Board of Kyungpook National University Dental Hospital (No. KNUDH-2021–07-03–00). Due to the retrospective design of this study using anonymized data, the Institutional Review Board of Kyungpook National University Dental Hospital waived the need for informed consent. All methods were carried out in accordance with relevant guidelines and regulations.

**Figure 1:**
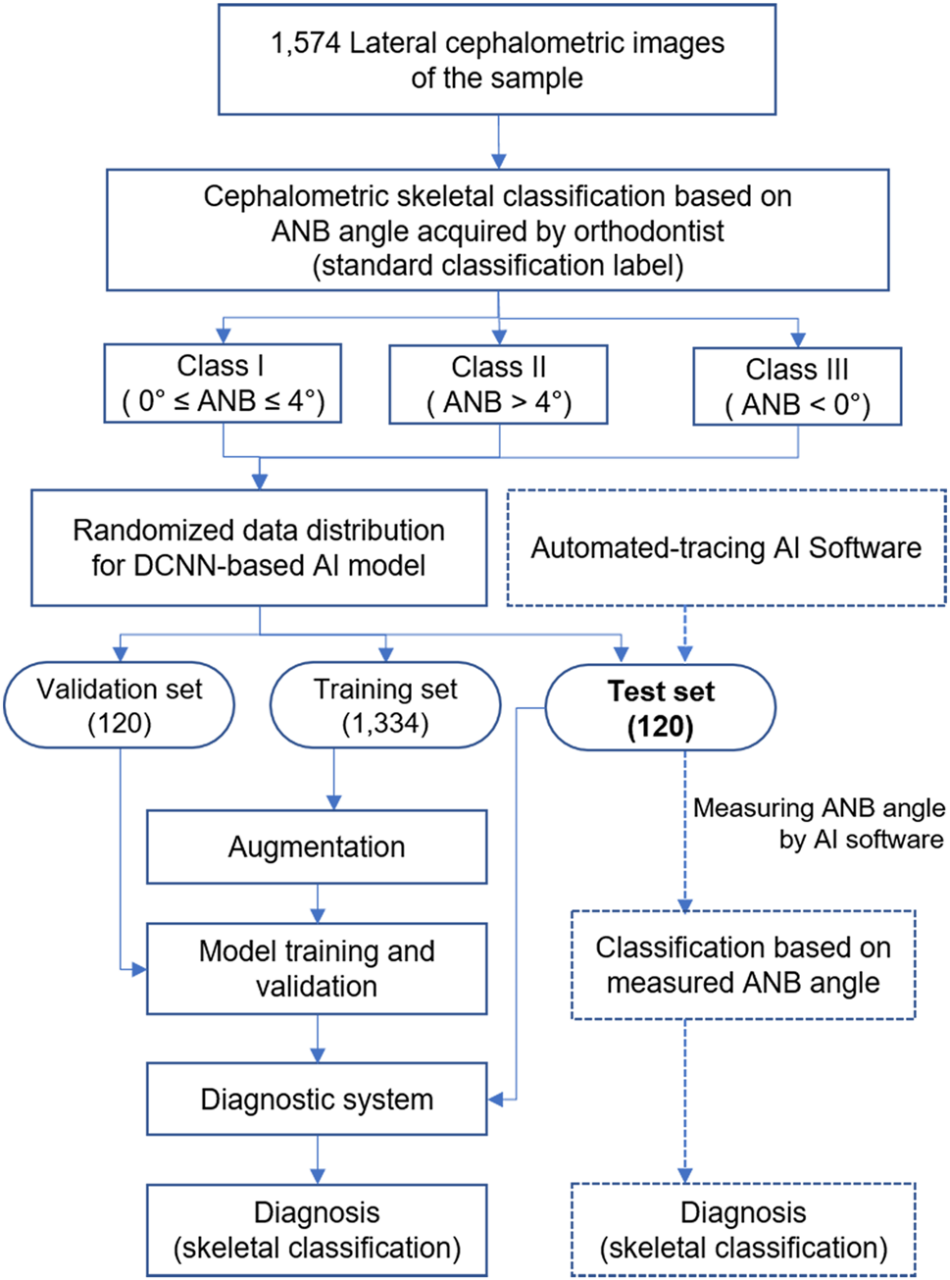
The flowchart of this study.

A total of 1,574 lateral cephalometric images of individual patients (745 males and 829 females with a mean age of 15.53 ± 8.14 years [range, 5.9–64 years]) who had undergone orthodontic diagnosis in the Department of Orthodontics at Kyungpook National University Dental Hospital in Daegu, Korea, from January 2012 to December 2020 were used (Fig. 1 and Table 1). All lateral cephalometric images were acquired using CX-90SP (Asahi, Kyoto, Japan) with a resolution of 5.91 pixels per millimeter. Patients with high-resolution lateral cephalometric images were included in this study. Prior to cephalometric classification, the points A (the most posterior point of the anterior concavity on the maxillary alveolar bone), B (the most posterior point of the anterior concavity on the mandibular alveolar bone), and N (the most anterior point of the frontonasal suture) were landmarked on the cephalometric image. Thereafter, the images were classified as skeletal Classes I, II, and III according to the ANB angle (angle between the NA and NB lines; Class I being 0–4°, Class II > 4°, and Class III < 0°). The landmark detection and skeletal classification were performed by a single examiner with 10 years of clinical orthodontic experience (HJK; standard classification label)^9^. The mean values of ANB angle were 2.3° in Class I, 6.6° in Class II, and –3.0° in Class III. All of the datasets were randomly divided into training, validation, and test sets including 1334, 120, and 120 images, respectively (Table 2). The training process was repeated 500 times with the training set. The test set of 120 images—40 images of each skeletal class I, II, or III relationship—was used to compare the performance of the DCNN-based AI model with that of the automated-tracing AI software (V-ceph, version 8.3, Osstem, Seoul, Korea). The AI software was developed using a dense convolutional network (DenseNet)—based deep learning algorithm and the edge AI concept^10,11^.

**Table 1:** Descriptive statistics of the sample in this study.

Parameters	Class I	Class II	Class III	Total
Total patients (n)	459	641	474	1574
Male	184	288	273	745
Female	275	353	201	829
Age (y, mean ± SD)	14.91 ± 7.56^A^	16.11 ± 9.50^A^	16.07 ± 7.27^A^	15.53 ± 8.14
ANB angle (°)	2.34 ± 0.75^A^	6.61 ± 1.48^B^	− 2.97 ± 2.08^C^	–

Values in the same row followed by the same letters are not significantly different at *p* < 0.05 according to a one-way analysis of variance with the post hoc Tukey's test.

**Table 2:** The number of patients assigned to training, validation, and test sets for deep convolutional neural network- (DCNN-) based AI model.

Date sets (n)	Class I	Class II	Class III	Total
Training	379	561	394	1334
Validation	40	40	40	120
Test	40	40	40	120
Total	459	641	474	1574

**Figure 2:**
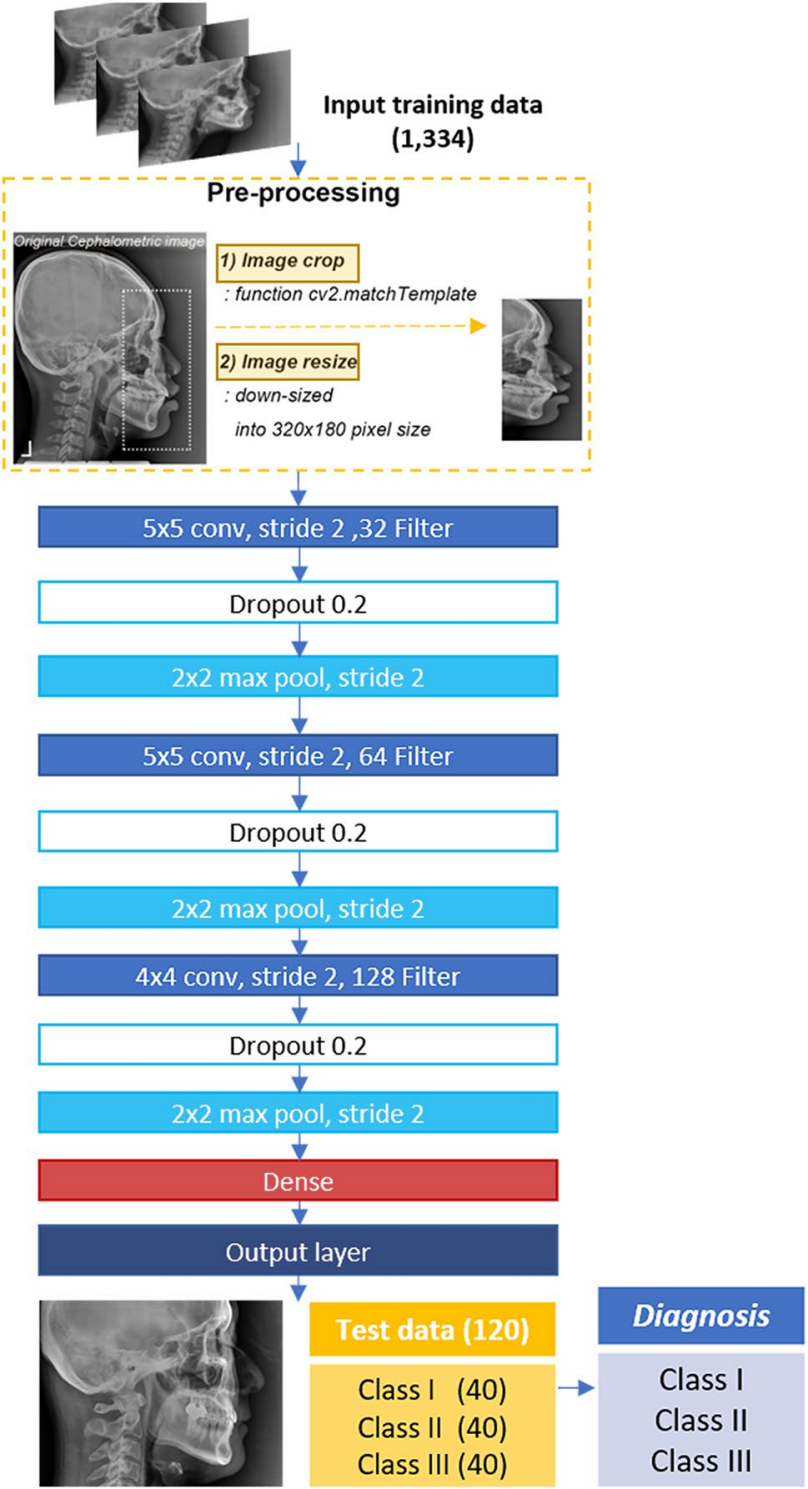
The process of deep convolutional neural network-based AI model used in this study.

As shown in Fig. 2., a new DCNN-based deep learning model was developed using the training data. For pre-processing the data, the image region involving A-, N-, and B-points (1500 × 800 pixels) was extracted from the original image (2460 × 1950 or 1752 × 2108 pixels) by performing template matching using the cv2.matchTemplate function (image cropping; Supplementary Fig. 1). Subsequently, the extracted images were down-sized into a 320 × 180-pixel size (image resize). To improve the performance of the model, data augmentation, such as rotating, shifting, or flipping images, and dropout were carried out. The learning rate was set to 0.001, the batch size to 64, and the number of epochs to 500. The accuracy and loss in training and validation were verified.

The age and ANB angle were compared between the three classes using a one-way analysis of variance with the post hoc Tukey's test, and a *p*-value of < 0.05 was considered statistically significant.

The agreement of the DCNN-based AI model or the automated-tracing AI software with a standard classification label was measured using Cohen’s kappa coefficient (< 0.00, poor; 0.00–0.20, slight; 0.21–0.40, fair; 0.41–0.60, moderate; 0.61–0.80, substantial; 0.81–1.00, almost perfect)^12^. Diagnosis in the skeletal classification of the DCNN-based AI model was achieved immediately, while the AI software diagnosis was based on the ANB angle derived from the three points detected automatically as mentioned above. To compare the performance of the DCNN-based AI model with that of the automated-tracing AI software, the sensitivity, specificity, precision, accuracy, and confusion matrix were evaluated on identical test sets.

## Results

### Descriptive statistics of the sample

There was no significant difference in ages between Classes I, II, and III (Table 1). The mean values of the ANB angle were 2.34°, 6.61°, and − 2.97° in Classes I, II, and III, respectively, showing a significant difference (*p* < 0.001).

### Performance of cephalometric skeletal classification for the DCNN-based AI model

Cohen’s kappa coefficient between the standard classification label and the DCNN model was in the range of 0.882 to 0.975, indicating almost perfect agreement (Table 3).

**Table 3:** Cohen’s kappa coefficients for agreement between the standard classification label and either DCNN-based AI model or automated-tracing AI software.

Test set	DCNN-based AI model		Automated-tracing AI software	
	Kappa	*p*-value	Kappa	*p*-value
Class I	0.975	0.000	0.905	0.000
Class II	0.975	0.000	0.975	0.000
Class III	0.882	0.000	0.720	0.000
Total	0.913	0.000	0.775	0.000

DCNN, deep convolutional neural network.

**Figure 3:**
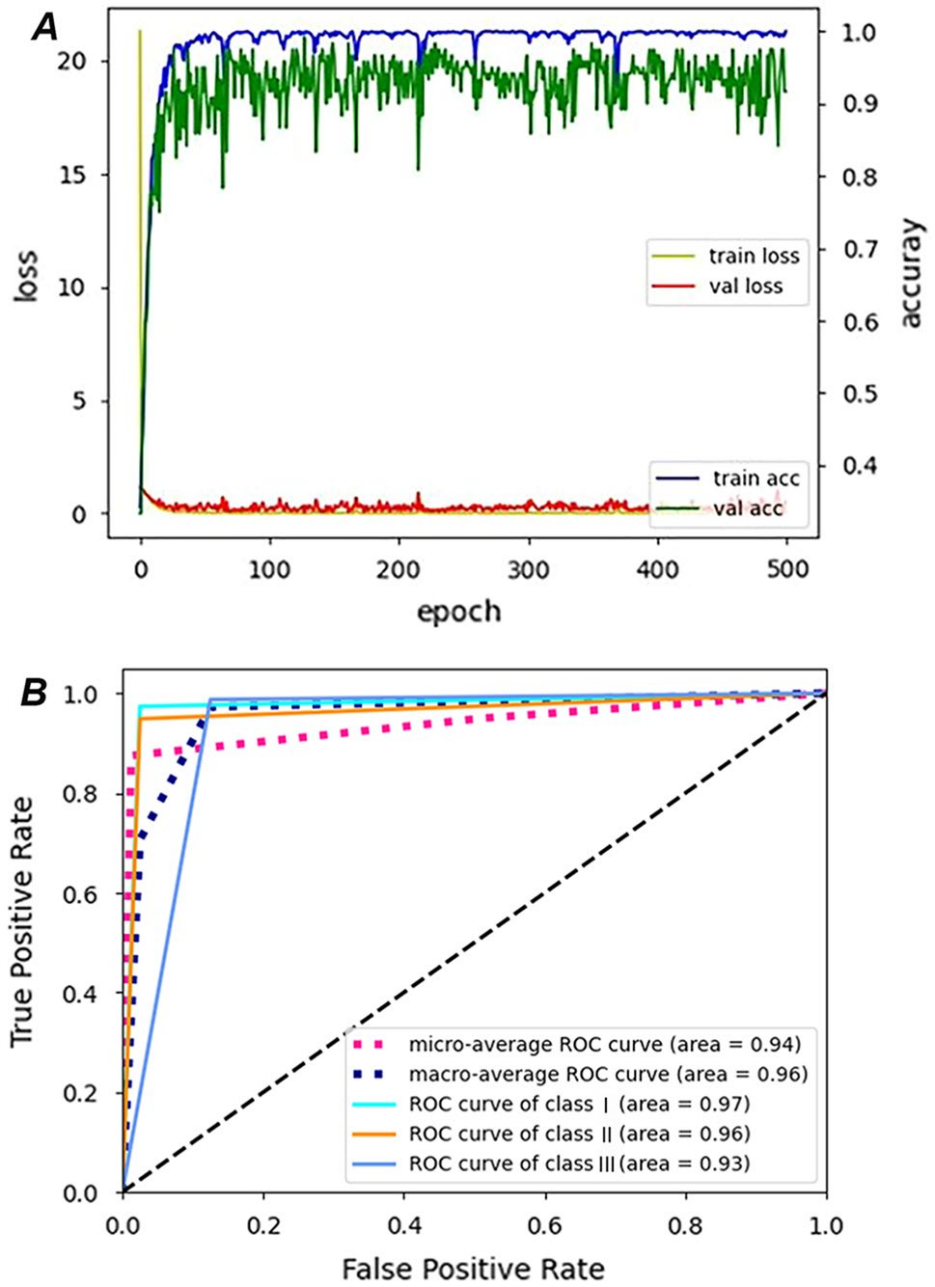
Performances of deep convolutional neural network-based AI model. A. Accuracy and loss of training and validation according to the number of epochs. B. Receiver operating characteristic (ROC) curve and the area under the curve (shown in parentheses).

**Figure 4:**
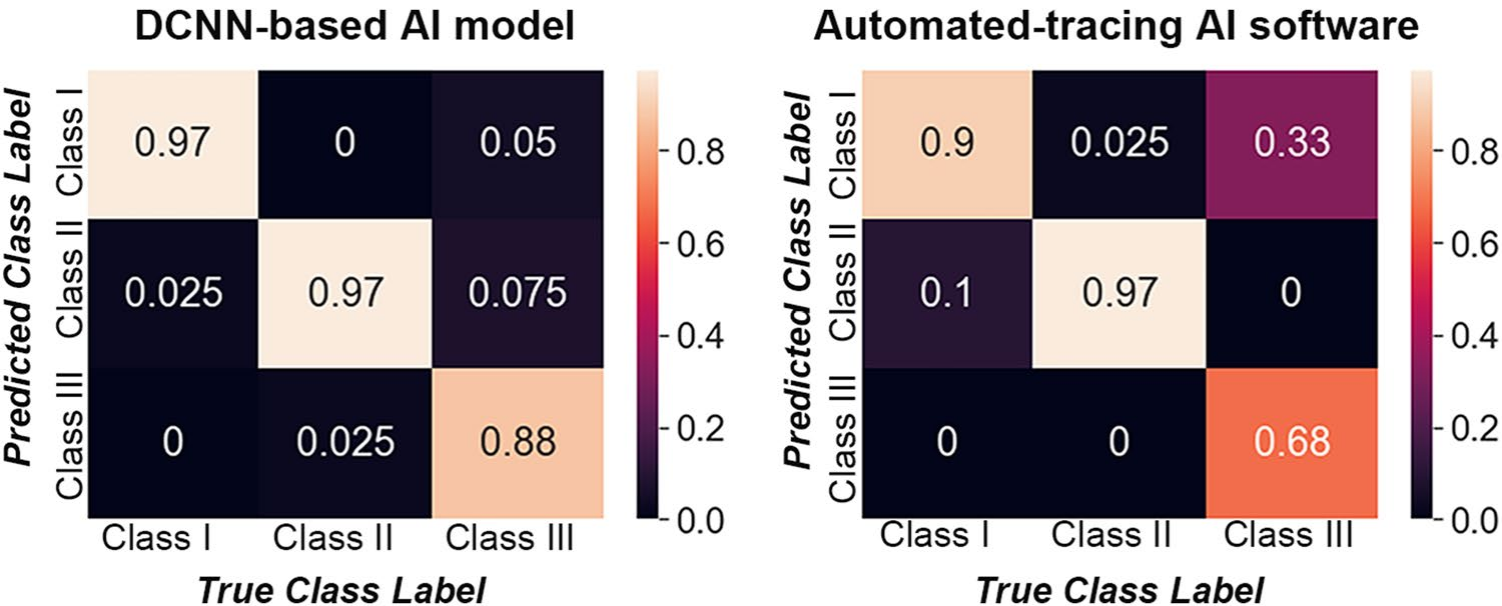
Confusion matrices of deep convolutional neural network- (DCNN-) based AI model and automated-tracing AI software.

Micro- and macro-average performance results included a sensitivity of 0.94, specificity of 0.97, precision of 0.94, and accuracy of 0.96 (Table 4). The accuracies of respective skeletal classes were 0.97 in Class I, 0.96 in Class II, and 0.95 in Class III. Figure 3A shows the accuracy and loss of training and validation according to the number of epochs. The receiver operating characteristic (ROC) curve represents the balance between sensitivity and specificity; a curve closer to the top-left corner of the graph indicates better performance (Fig. 3B). The area under the ROC curve (AUC) is an effective method for explaining the overall accuracy of the DCNN-based AI model. AUC takes values between 0 and 1, which a value of 0 or 1 indicates a completely inaccurate or completely accurate model, respectively^13^. In this study, the AUC (micro-average ROC curve) was 0.94, indicating 94% probability that the DCNN model will correctly execute the skeletal classification based on the cephalometric images. In the confusion matrix of the DCNN model, the correct predictions in Classes I and II were higher than in Class III (Fig. 4).

**Table 4:** Performances of cephalometric skeletal classification for DCNN-based AI model and automated-tracing AI software.

	DCNN-based AI model				Automated-tracing AI software			
	Sensitivity	Specificity	Precision	Accuracy	Sensitivity	Specificity	Precision	Accuracy
Class I	0.97	0.98	0.95	0.97	0.90	0.83	0.72	0.85
Class II	0.97	0.95	0.91	0.96	0.98	0.95	0.91	0.96
Class III	0.88	0.99	0.97	0.95	0.68	1.00	1.00	0.89
Micro-average	0.94	0.97	0.94	0.96	0.85	0.93	0.85	0.90
Macro-average	0.94	0.97	0.94	0.96	0.85	0.93	0.90	0.88

DCNN, deep convolutional neural network.

**Figure 5:**
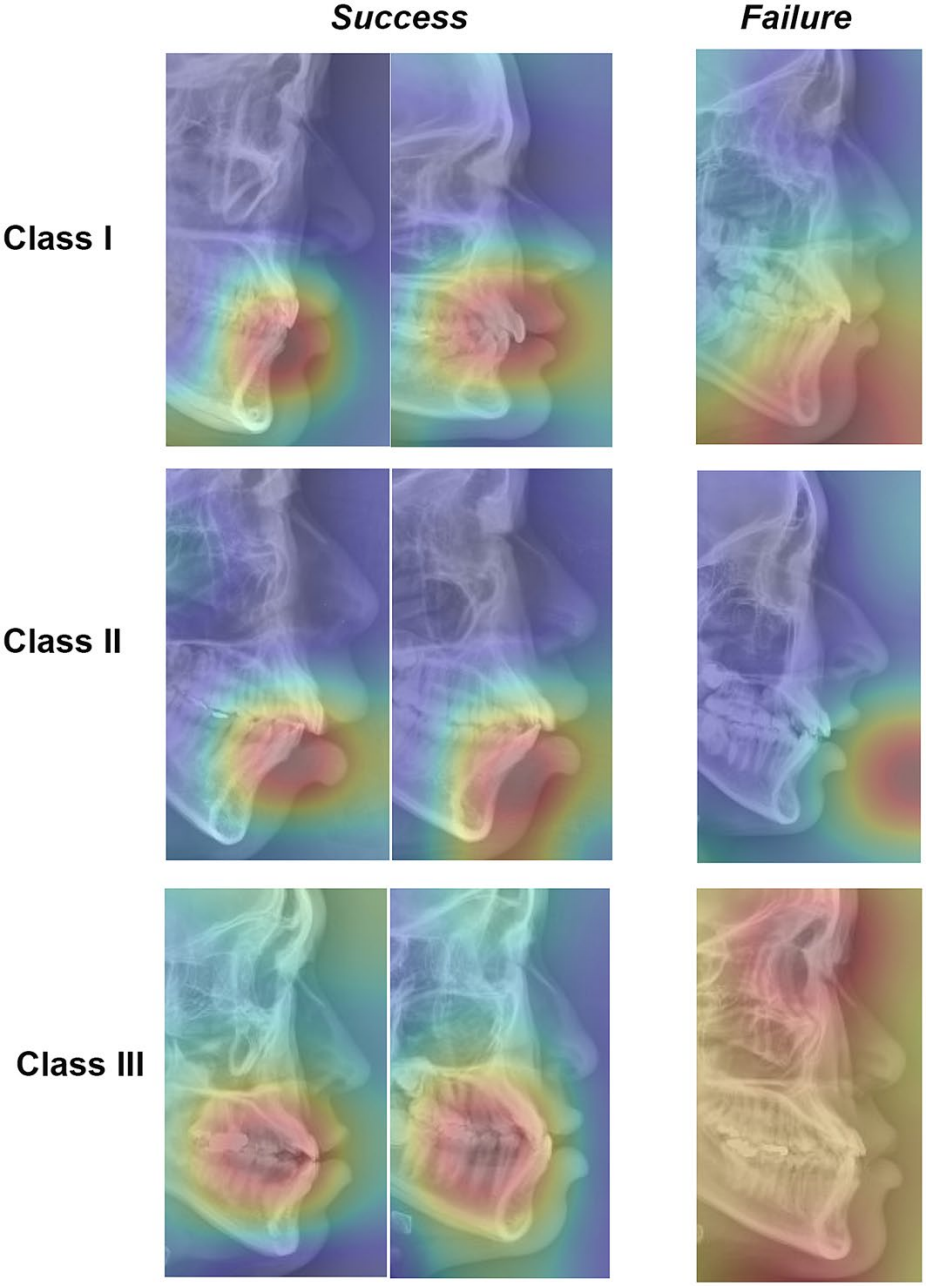
Class activation maps showing the regions of interest (ROI) of success and failure images (by the current DCNN model).

The current DCNN algorithm correctly classified the images, with the regions of interest (ROI) placed on the A- and B-points, anterior teeth, and upper and lower lips (Fig. 5). In contrast, in the case of the failed predictions, the ROI was indistinct, widespread, and/or focused on irrelevant structures.

### Performance of cephalometric skeletal classification for the automated-tracing AI software

Regarding classification agreement, Cohen’s kappa coefficient between the standard label of classification and the AI software varied from 0.720 to 0.975, which can be interpreted as substantial to almost perfect agreement (Table 3).

When evaluating the performance of classification using the automated-tracing AI software, the micro-average values had a sensitivity of 0.85, specificity of 0.93, precision of 0.85, and accuracy of 0.90 (Table 4). The accuracies of each class were 0.85 in Class I, 0.96 in Class II, and 0.89 in Class III. As shown in Fig. 4, based on the confusion matrix, Class III images exhibited a lower success rate in skeletal diagnosis than Class I and II images.

## Discussion

In orthodontics, research on deep learning algorithms is being increasingly conducted. The well-known and promising topics include automated cephalometric landmark identification^3,14,15^, classification or diagnosis for treatment planning^6–8,16,17^, and tooth segmentation and setup using three-dimensional digital tools such as cone-beam computed tomography (CBCT) and scan data^18,19^.

In particular, DCNN algorithms demonstrating a robustness in medical image analysis are clinically helpful in reliable decision-making and obtaining an accurate diagnosis. Hence, in this study, the new DCNN-based AI model was developed and examined for sagittal skeletal classification using lateral cephalometric images. The extracted images including A-, B-, and N-points effectively helped the model training as part of pre-processing. When sampling the cephalometric images, all images with good resolution were included irrespective of dental prosthesis, implant, age, and even history of cleft lip and palate. The diverse images might be associated with higher performance of the current DCNN model compared with that of the other DCNN models from the earlier studies as well as the AI software^6,8^. Proper neural network depth might be another factor leading to better performance, as observed in this study^4^.

A class activation mapping (CAM) is fairly useful in visualizing the discriminative image regions when assessing the ROI used by the current DCNN models^20^. In this study, although the N-point was not indicated by the CAM, A- and B- points were commonly highlighted in the successfully classified images.

**Figure 6:**
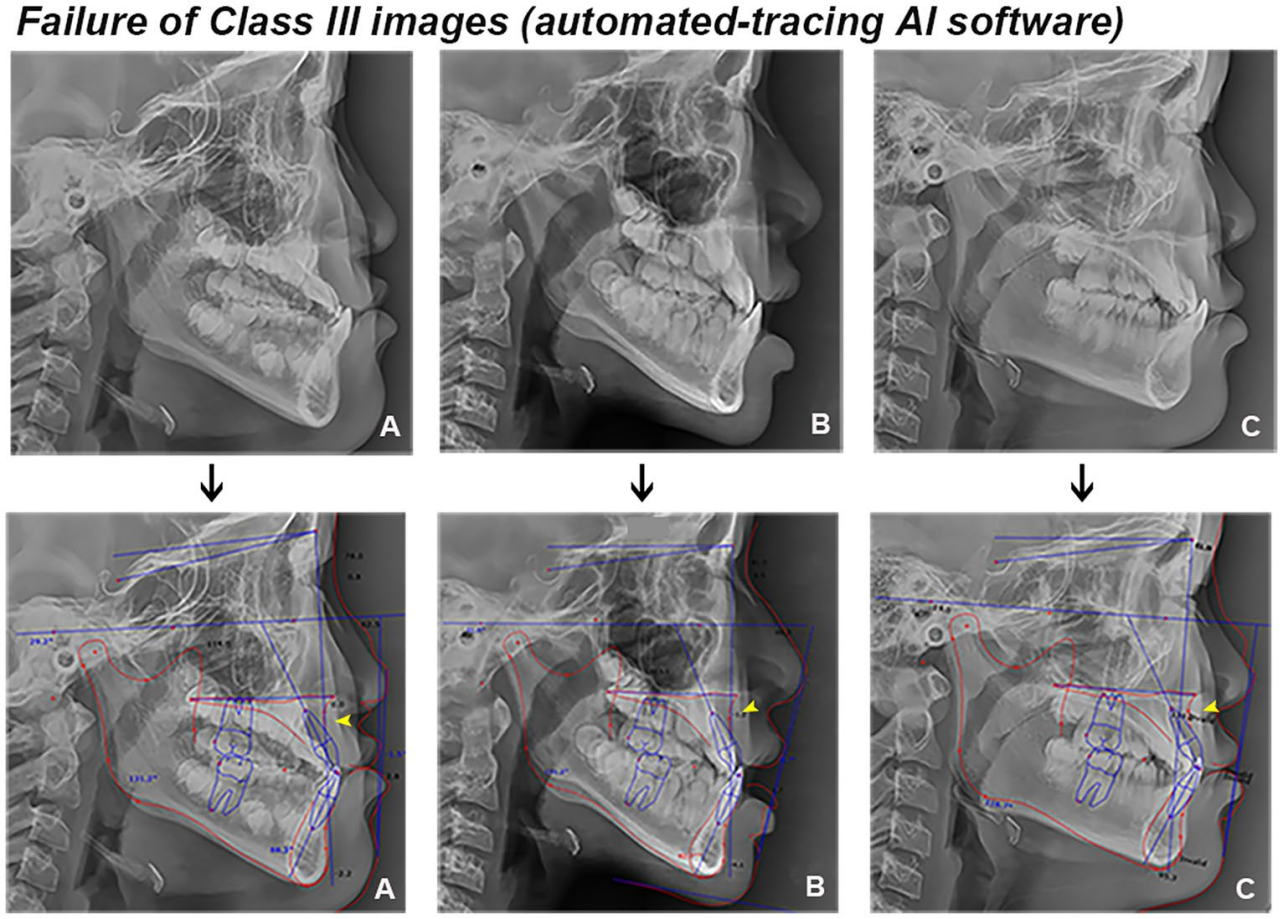
Examples of Class III images of automated-tracing AI software (*arrow*, erroneous detection of A-point).

Meanwhile, regarding the automated landmark detection method, the success rate of detection has improved through the previous research^21,22^. Recently, Lee et al.^23^ reported a mean landmark error of 1.5 mm and a successful detection rate of 82% in the 2 mm range, and Hwang et al.^13^ highlighted detection errors < 0.9 mm compared to human results, indicating that automated detections were clinically acceptable. Despite these gradual improvements in the detection accuracy of AI, pin-pointing a particular landmark is not straightforward even for an experienced orthodontist. Specifically, the A- and B-points used in this study are in general well-known for being error-prone during detection. In a previous study on automated landmark identification, detection errors of 2.2 mm for the A-point and 3.3 mm for the B-point were higher than the mean value of 1.5 mm in all landmarks^13^. Yu et al^2^ also mentioned the difficulty in identifying the A-point of cephalometric analysis based on AI. In this study, the automated-tracing AI software often identified the two points erroneously, which likely led to the rather lower performance compared with the DCNN model. Furthermore, an interesting finding is that the sensitivity—the ability of a test to correctly identify the skeletal classification (true positive rate) —of the AI software on Class III images was far lower than that of other classes (Fig. 4 and Table 4). As presented in Fig. 6, the thicker lip soft tissue around the A-point in Class III patients likely led to more inaccurate identification of the landmark^24,25^, and this might be rather enhanced in patients with cleft lip and palate^26^. In this regard, compared with the AI software that pin-pointed the landmarks, the DCNN model with a larger ROI might show better performance in skeletal classification.

Although it is challenging to compare these two AI models in a straightforward manner, it would be worth investigating the performances for precise diagnosis and decision-making. The newly developed image-based DCNN algorithm enables clinicians to directly achieve accurate diagnoses and predict treatment outcomes. Thus, it can provide valuable opinions with regard to decision-making and treatment planning without the time-consuming process of cephalometric landmarking and analyzing. Nonetheless, a precise analysis using the landmarks of cephalogram is critical to determine the degree of skeletal and dental discrepancy and obtain other informative measurements. In particular, some variables can be weighted to impact the orthodontist’s decision in treatment planning.

Although the current study has successfully investigated the DCNN-based AI model and compared the two AI models for skeletal classification, there is a limitation in the availability of heterogeneous learning data for the two respective AI algorithms. In addition, as mentioned by a previous study^27^, a combination of various measurements or variables leads to better performance in sagittal skeletal classification than using a single ANB angle. Therefore, orthodontic analysis is required in patients with sagittal, transverse, and/or vertical problems using multi-source data, such as facial and intraoral scan data, CBCT images, and demographic information, along with a more advanced algorithm model.

It would be interesting to investigate the performance of the DCNN model in predicting facial growth using cervical vertebrae maturation and/or hand-wrist radiographs and to further evaluate the relationship between the predictions.

## Conclusion

With regard to skeletal classification using lateral cephalometric images, the performance of the current DCNN-based AI model was better than that of the automated-tracing AI software. The DCNN model might be useful in clinical practice in terms of providing objective and valuable second opinions for skeletal diagnosis of cephalometric images.

## Supplementary Information

Supplementary Information.

## Data Availability

The datasets generated and/or analyzed during the current study are not publicly available as the confidential information therein may compromise patient privacy and violate the ethical policies of our institution but are available from the corresponding author on reasonable request. Data usage agreements may be required.
