# Supplementary material for: Deep convolutional neural network-based skeletal classification of cephalometric image compared with automated-tracing software
Source: Sci Rep. 2022 Jul 8;12:11659. doi: 10.1038/s41598-022-15856-6 (PMC9270345; doi:10.1038/s41598-022-15856-6)
Supplement: Supplementary file 1 — Supplementary Information. [file 41598_2022_15856_MOESM1_ESM.docx]

**Deep convolutional neural network-based skeletal classification of cephalometric image compared with automated-tracing software**

**Ho-Jin Kim*^1^; Kyoung Dong Kim^2^; Do-Hoon Kim^3^**

^1^ DDS, MSD, PhD, Assistant Professor, Department of Orthodontics, School of Dentistry, Kyungpook National University, Daegu, Korea.

^2^ MS, Graduate student, School of Electronic and Electrical Engineering College of IT Engineering, Kyungpook National University, Daegu, Korea.

^3^ MD, PhD, Associate Professor, Medical Big Data Research Center, Kyungpook National University, Daegu, Korea.

**Corresponding Author:** Dr. Ho-Jin Kim, Assistant Professor, Department of Orthodontics, School of Dentistry, Kyungpook National University, 2175, Dalgubul-Daero, Jung-Gu, Daegu 41940, Korea

E-mail: [hojinkim@knu.ac.kr](file:///C:\Users\SJLindauer\Downloads\hojinkim@knu.ac.kr)


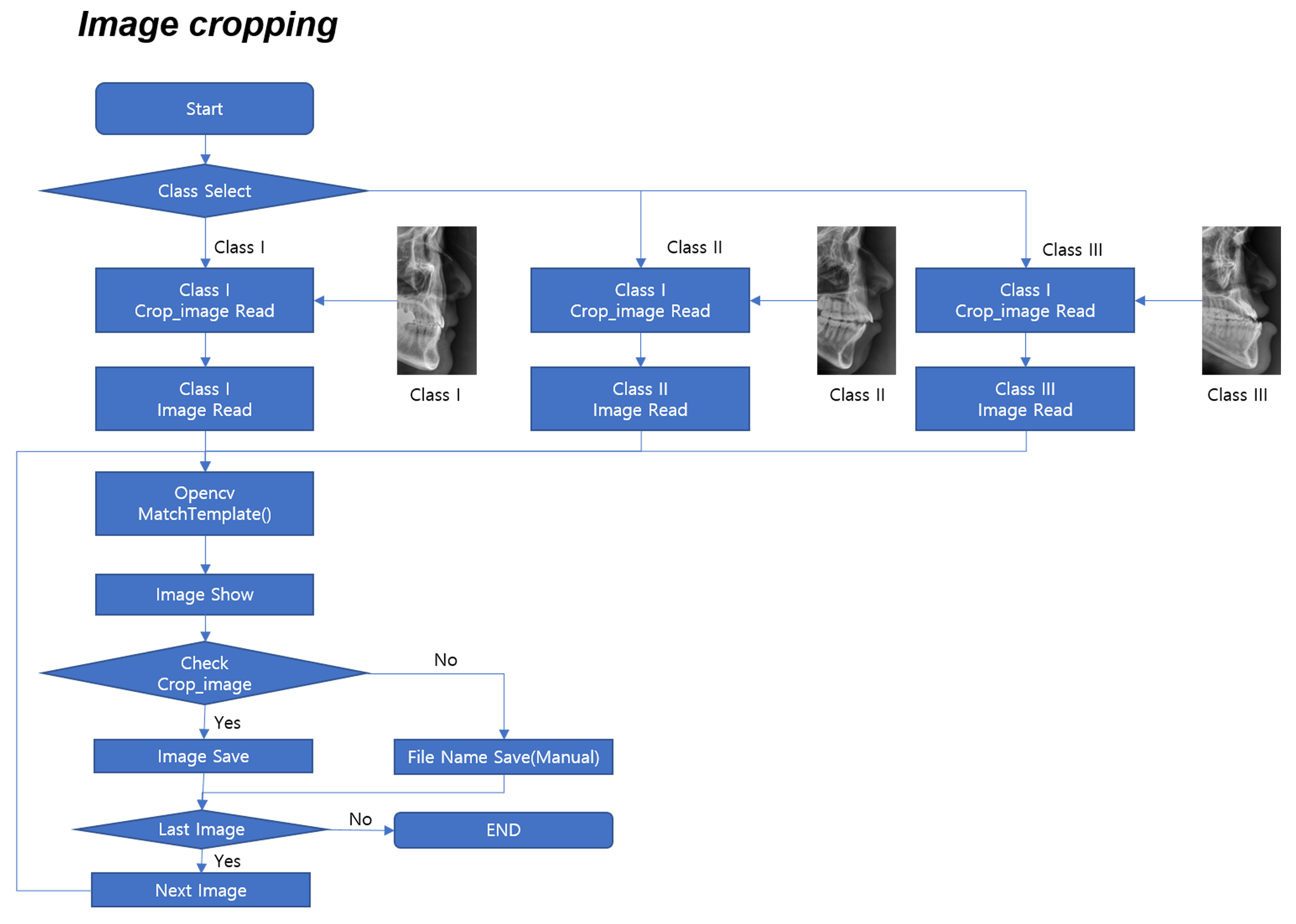


Supplementary Fig. The flow chart of the image cropping.
